# Aging of Attentiveness in Border Collies and Other Pet Dog Breeds: The Protective Benefits of Lifelong Training

**DOI:** 10.3389/fnagi.2017.00100

**Published:** 2017-04-20

**Authors:** Durga Chapagain, Zsófia Virányi, Lisa J. Wallis, Ludwig Huber, Jessica Serra, Friederike Range

**Affiliations:** ^1^Clever Dog Lab, Messerli Research Institute, University of Veterinary Medicine Vienna, Medical University of Vienna, University of ViennaVienna, Austria; ^2^Department of Ethology, Eötvös Loránd UniversityBudapest, Hungary; ^3^Royal Canin Research CentreAimargues, France

**Keywords:** aging, training, pet dogs, attentional capture, sustained attention, selective attention

## Abstract

Aging of attentiveness affects cognitive functions like perception and working memory, which can seriously impact communication between dogs and humans, potentially hindering training and cooperation. Previous studies have revealed that aged laboratory beagles and pet Border collies (BC) show a decline in selective attention. However, much less is known about the aging of attentiveness in pet dogs in general rather than in specific breeds. Using 185 pet dogs (75 BC and 110 dogs of other breeds) divided into three age groups [late adulthood (6- < 8 year), senior (8- < 10 year) and geriatric (≥10 year)], we assessed the progress of aging of attentional capture, sustained and selective attention in older dogs in order to explore if prior results in BC are generalizable and to evaluate the influence of lifelong training on measures of attention. Each dog’s lifelong training score (ranging from 0 to 52) was calculated from a questionnaire filled in by the owners listing what kinds of training the dog participated in during its entire life. Dogs were tested in two tasks; the first, measuring attentional capture and sustained attention toward two stimuli (toy and human); and the second, measuring selective attention by means of clicker training for eye contact and finding food on the floor. In the first task, results revealed a significant effect of age but no effect of lifelong training on latency to orient to the stimuli. Duration of looking decreased with age and increased with lifelong training. In the second task, while lifelong training decreased the latency of dogs to form eye contact, aged dogs needed longer to find food. BC did not differ from other dogs in any measures of attention except latency to find food. In conclusion, aged dogs showed a decline in attentional capture and sustained attention demonstrating that these tests are sensitive to detect aging of attentiveness in older pet dogs. Importantly, selective attention remained unchanged with age and lifelong training seemed to delay or reduce the aging of attentiveness, further highlighting the importance of lifelong training in retaining general cognitive functions.

## Introduction

Aging leads to significant changes in the brain which progressively impair cognition, behavior and the quality of life (Youssef et al., 2016). The impairment of different cognitive functions include decline in learning and memory, maintaining focused attention on a task, inhibiting responses to certain irrelevant stimuli and many more (Quigley et al., 2012; Zanto and Gazzaley, 2014). The decline in attention has been found to be particularly critical due to its essential role for goal directed behaviors and other perceptual and cognitive functions (Tipper, 1991; Staub et al., 2013).

Attention has been proposed to consist of multiple components that interact during cognitive functioning (Cornish et al., 2007) with sustained attention and selective attention being widely studied in humans (Sarter et al., 2001; Mani et al., 2005; Staub et al., 2013; Geerligs et al., 2014). Sustained attention (as measured by the ability to achieve and maintain focus on a given stimulation source or task) declines as aging progresses in humans (Staub et al., 2013). However, there are some studies reporting conflicting results with either alteration or preservation of sustained attention during aging (Berardi et al., 2001; Mani et al., 2005; Staub et al., 2013; Zanto and Gazzaley, 2014). Findings on sustained attention tasks in elderly humans with age related dementia also differ, with some showing impairments at an early or an advanced stage of the disease, or no impairment at all (Staub et al., 2013). In contrast, results on selective attention [as measured by the ability to focus on task-relevant goals to the exclusion of salient distracters (Matzel and Kolata, 2010)] have indicated age related decline and severe deterioration in people suffering from dementia (Sarter et al., 2001; Staub et al., 2013).

Studies examining aging of attention in dogs have found similar results to that of humans (Snigdha et al., 2012; Wallis et al., 2014). Wallis et al. (2014) reported stimulus difference in sustained attention, with no difference between younger (>6 months) and older (>10 years) individuals when sustaining attention to a social stimulus in clear contrast to a non-social stimulus where an attention deficit in older individuals was found. For the measures of attentional capture, a quadratic effect of age was documented reflecting lower attention in younger and older individuals. Selective attention, as measured by presenting dogs with a task switching paradigm where dogs had to make eye contact with the experimenter and find food on the floor, also showed a quadratic relationship with age. Snigdha et al. (2012) utilized a visual search task to measure selective attention in laboratory beagle dogs and reported that senior dogs were significantly impaired in accuracy and reaction time compared to younger dogs, reflecting deficits in selective attention. Other studies have focused on the characterization of the changes of attention in adult and older dogs in a social context (Range et al., 2009; Mongillo et al., 2010). Mongillo et al. (2010) measured selective attention toward the owner and a stranger by presenting them simultaneously to the dogs. Older dogs were less able to discriminate between the owner and the stranger, and hence looked longer at the stranger compared to adult dogs, displaying a decline in selective attention. These studies have highlighted how different attention parameters change during the aging process in dogs, and pose questions regarding possible factors and interventions which can influence the aging of attention either positively or negatively.

Interventions like physical activity and cognitive training are associated with improved attention and executive control processes such as task switching and inhibition in older humans (Shatil, 2013). A number of studies support the beneficial effects of training, both in task training and lifelong training experiences on the physical and cognitive health of older humans, assessed by measuring different cognitive functions, including attention (Churchill et al., 2002; Colcombe et al., 2003; Chang et al., 2012; Shatil, 2013; Berchicci et al., 2014; Bullock and Giesbrecht, 2014). Oswald et al. (2006) reported that combined training (physical activity and cognitive training) had a major benefit to older humans’ cognitive function, physical function, health status, emotional status and wellbeing, and furthermore, this training effect endured even 5 years after intervention. Thus, the results of many cross-sectional, prospective, and retrospective epidemiological studies in humans have suggested a link between physical activity and cognitive benefits to old adults (for details see Churchill et al., 2002). Similar to humans, Milgram and colleagues in numerous studies have documented benefits of physical activity and cognitive enrichment on the performance of laboratory dogs in different cognitive tasks (Milgram et al., 2002, 2004, 2005; Milgram, 2003). In these studies, they used physical activity and cognitive enrichment as an intervention and assessed their effect on learning and memory task. But there are studies in pet dogs that have used dogs’ lifelong training experiences documented via owner reported questionnaire instead of in task training, and assessed its effect on different cognitive tasks (Marshall-Pescini et al., 2008, 2009, 2016; Range et al., 2009). The results of these studies demonstrated that those dogs with high lifelong training score performed better than novice dogs. Additionally, studies on laboratory beagles have also documented the benefits of prior cognitive experience on discrimination learning task (Milgram, 2003). Therefore similar positive effects of lifelong training on attention parameters could be expected in dogs. Overall, while we have some evidence of positive effects of lifelong training experiences on attention in humans (Churchill et al., 2002; Berchicci et al., 2014) and benefits on cognitive tasks in dogs, we lack sufficient reports of a positive effect of lifelong training, particularly on measures of attention in pet dogs.

Moreover, even less is known about aging of attentiveness in pet dogs in general rather than in specific breeds like laboratory beagles and Border collies (BC). The results obtained in laboratory beagles can only be generalized to a limited extent because of the homogeneity of subjects with respect to breed and cognitive experiences, the extensive training required for testing and the small sample sizes used in these studies (Cory, 2013). Regarding BC, it is a special breed often with owners who engage their dogs in different activities on a daily basis and spend a lot of time undertaking dog training. In Wallis et al. (2014) study, the Border collie owners participated in on average five different types of training and spent 6 h per week doing different types of physical and mental training. Therefore, this breed possibly represents a more highly trained than the average pet dog and may behave differently than dogs of other breeds, which needs further investigation.

In order to more closely examine the aging of attention in pet dogs, and to address the question whether the aging trajectories of various components of attention are similar in other breeds compared to those found in pet BC, we tested aged pet dogs (>6 years) of different breeds (including mixed breeds). We investigated whether we can extend the previous findings in BC to different breeds using a similar setup and tests (for details see: Wallis et al., 2014). We also examined whether lifelong training experiences had any effect on the aging of attention. Based on previous findings in BC and humans, we predicted that all the measured components of attention – attentional capture, sustained and selective attention will decline with age, with geriatric dogs showing the poorest performance. Moreover, we predicted no differences in the aging patterns of various breeds as compared to BC, and finally, that lifelong training can hinder the aging of attentiveness.

## General Methods

### Ethics Statement

The institutional ethics and animal welfare committee at the University of Veterinary Medicine Vienna (Protocol number: 05/03/97/2014) and Royal Canin ethical committee (21/07/2014) approved this experiment. All dog owners signed a consent form prior to testing.

### Subjects

Seventy five BC and 110 dogs of other breeds including mixed breeds participated in the study. Fifty-nine BC were tested by Wallis et al. (2014); we added 16 new BC to this sample. The newly tested 16 BC did not differ from 16 BC (matched in age, sex, and lifelong training score) tested in the previous study in any of the variables analyzed in the experiments below (for details see Supplementary Material). The dogs’ age ranged from 6.2 to 14.2 years (74–170 months). They were divided into three age groups: late adulthood (6- < 8 years), senior (8- < 10 years) and geriatric (≥10 years) dogs (**Table 1**). The average weight for BC was 20.8 kg (range: 16–25 kg) and 21.9 kg (range: 7–42 kg) for other breeds. All dogs recruited for the study were kept as family pets and none had been referred for behavioral consultations at the time of recruitment. Dogs that were reported by their owners (via questionnaire) as not medically fit, including suffering from eye abnormalities and any detrimental medical condition (other than behavioral or cognitive effects of old age) were not included.

**Table 1:** Age, sex, reproductive status and lifelong training score of subjects.

Dog breed	Age group	Age in years	Age in months Mean ± SD	Male (intact)	Female (intact)	Total	Lifelong training score
	Group1	6- < 8	84.5 ± 7.52	14 (5)	11 (2)	25	15.76 ± 5.46
	(Late adulthood)						
Border collies	Group2 (Senior)	8- < 10	106 ± 6.60	14 (6)	12 (2)	26	16.46 ± 8.57
	Group3 (Geriatric)	≥10	138.1 ± 11.64	9 (3)	15 (1)	24	15.16 ± 4.36
			Average age: 109.36 ± 23.55				Average score: 15.94 ± 6.31
	Group1	6- < 8	83.40 ± 6.17	17 (6)	15 (3)	32	10.50 ± 8.72
	(Late adulthood)						
Other breeds	Group2 (Senior)	8- < 10	106.12 ± 6.34	8 (5)	24 (3)	32	12.53 ± 7.95
	Group3 (Geriatric)	≥10	138.95 ± 14.36	24 (3)	22 (1)	46	12.26 ± 8.70
			Average age: 113.24 ± 25.74				Average score: 11.84 ± 8.50
							***p* < 0.001^a^**

*^a^Significant difference between dog breeds irrespective of age_groups (Linear model).*

The two tests presented here were part of the modified version of the “Vienna Canine Cognitive Battery” (VCCB) consisting of 10 different tests in which all dogs participated. Since the first session of the VCCB preceded the two attention tests presented, all dogs had visited the lab at least once prior to the tests and had experience of working with the experimenter (E). During recruitment, owners filled in an extensive demographic questionnaire including questions on their dog’s past and current attendance to 13 different types of training (Puppy school, obedience, agility, BGH, protection dog training, service dog training, search and rescue training, dog dancing/trick training, dummy training, hunting/nose work, sheep dog training, therapy dog training, others). Each kind of training was scored as follows: no experience = 0, sporadic training = 1, once or twice a month = 2, once or twice a week = 3 and completed training (with or without an exam) = 4. Based on the 13 scores, a lifelong formal training score (ranging from 0 to 52) was calculated by summing up all the scores. Previous clicker training experience (yes/no) of the dogs was available from the questionnaire.

### Test Setting

Both tests were conducted in the same experimental room (7.12 m × 6 m) by the same experimenter (E). The room was empty with the exception of a chair for the owner, a small orange plastic watering can (children’s toy) hanging from the ceiling and four digital video cameras connected to a video-recording station outside the test room. The toy was attached to a fishing line that ran through a metal hoop on the ceiling, allowing the toy to be manipulated from outside the room during the testing. E, while sitting outside the room, could see what was going on in the room on the monitor of the video recording station. During the task, owners were instructed to ignore their dog and to be quiet and not move. All owners followed the instructions and did not attempt to interact with their dogs.

### Data Collection and Statistical Analyses

Tests were recorded using a set-up of four digital video cameras inside the room. The videos generated from the tests were later coded and analyzed using the video-coding software Solomon Coder beta 15.11.19 (by Andras Peter^1^) with a continuous sampling technique. All statistical analyses were performed in R 3.2.2 (RStudio 2015) and the graphical illustrations were done in IBM SPSS statistics V24. Separate statistical models were calculated first with age as a continuous variable and then with age as a categorical variable to look for specific differences across age groups. Normality and homoscedasticity were assessed via residual distribution charts and plots of residuals against fitted values. Non-significant predictors (*p* > 0.05) were removed from the model and are not reported in the results. Results from the comparison of age groups were Bonferroni corrected for multiple comparisons and are considered significant at *p* < 0.05. Results are presented as mean ± standard deviation unless otherwise indicated. Extreme outliers detected in the model were removed, and the models were re-run as sensitivity analyses. Before running the model analyses, we checked for a correlation between age in months and lifelong training score and found no correlation (Spearman correlation: *r* = 0.02, *p* = 0.78). Therefore, we used both age in months and lifelong training score as covariates in each model. The size of the effect for the significant and non-significant results were calculated using *R*^2^ as suggested by Durlak (2009) and Nakagawa and Schielzeth (2013). In addition to running different statistical models, we used equivalence tests (TOST) to validate the difference between the two group means (Border collie and other breeds) in Excel using the XLSTAT software. While the purpose of a traditional hypothesis test is to determine whether two groups differ from one another, equivalence analysis is used to determine whether two groups are sufficiently similar to each other to be considered equivalent. Equivalence analysis is a method for estimating whether a difference between groups, if one exists, is smaller than a predetermined threshold. In this analysis, both Type I and Type II error rates are controlled. The first step in equivalence testing is to define the range (threshold) used for comparison and then to perform two simultaneous one sided hypothesis tests (Rogers et al., 1993). Although, there is a discrepancy regarding the threshold between a negligible and a useful effect, the best accepted method is to use a percentage of the mean of the control group, where a 5% difference between group means might be considered a conservative standard for establishing equivalence, while a 20% difference might be considered liberal (Rogers et al., 1993). Because there is no relevant predetermined guideline, an equivalence of 10% was used for this analysis, and the Border collie group used as the control group. We ran Fisher’s *F*-test (two-tailed) in order to determine whether the variances for the two sets of data are equal or not. For those variables with unequal variances, we used the Cochran-Cox approximation when computing the critical value of the *t* statistic and *p*-value.

## Experiment 1: Attention Test

In Experiment 1, using a similar paradigm as Wallis et al., (2014), we assessed whether attentional capture and sustained attention, measured by the latency and duration of looking at the stimuli (toy and human) respectively, were affected by age, breed group and lifelong training. Wallis et al. (2014) found an effect of age in attentional capture and, in the non-social task in sustained attention in 145 BC. Similarly, a study on rhesus monkeys indicates a decline of sustained visual attention in older animals compared to younger ones using a touchscreen task (Zeamer et al., 2011). Based on these previous research findings, we predicted that attentional capture and sustained attention will decline with age, with geriatric dogs showing the greatest decline in performance.

### Procedure

The owner entered the room with their dog, attached it by the collar to a 1.5 m leash fixed to the wall, sat down on a chair facing away from the dog (toward the window) and pretended to read the protocol. Two conditions were presented in a counterbalanced order to each dog: a non-social condition and a social condition.

#### Non-social Condition

**FIGURE 1:**
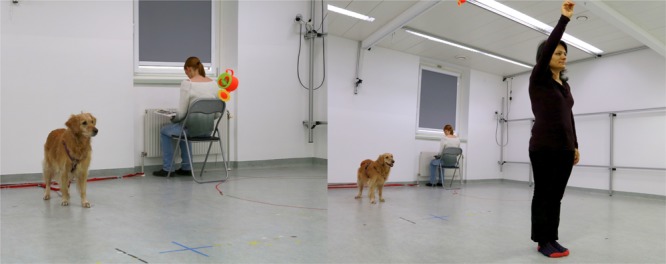
**Dog watching the non-social stimulus (flying toy) and the social stimulus (moving human)**.

After the owner and the dog were in position, E pulled a fishing line from outside to lift the toy up from the floor and then moved it up and down in the center of the room. By watching the dog on the outside screen, E made sure that she started to move the toy when the dog was looking away from it. The toy was moved up and down in front of the dog for 1 min (**Figure 1**). After 1 min, E fixed the toy to the ceiling and signaled the owner to come outside with the dog by saying “okay.”

#### Social Condition

After the owner and the dog were in position and the dog looked away from the door, E entered the testing room, closed the door, walked to a blue cross on the floor marked at a distance of 3 m from the dog and started to move up and down vertically pretending to paint an invisible wall for 1 min (**Figure 1**). While moving, she had her back to the dog so that the dog had no chance to establish eye contact with her. After 1 min, E went outside and then signaled the owner to leave the room with the dog by saying “okay.” In order to improve the methods of our previous paper (Wallis et al., 2014), we slightly changed social condition. In the previous paper, after entering the room, E walked up and down along the wall (6 m) with a paint roller in her hand, meaning that in the previous study (Wallis et al., 2014), the movement of the toy was vertical whereas the human moved in a lateral direction. In the current study, the movement of both stimuli was more comparable, i.e., vertical. Otherwise the methods of the two studies were identical. All 110 dogs of different breeds and the 16 new BC were tested with this new methodology.

### Behavioral and Statistical Analyses

Attentional capture was measured as the latency to orientate to the stimuli [measured from the first detectable movement of the toy/door handle up to the point where the dog’s gaze (head and nose) was centered upon the stimulus]. Sustained attention was measured by calculating the duration of looking at the stimulus (toy/human). A randomly chosen set of 20 dogs were coded by a second coder, and inter observer reliability was calculated by estimating the intraclass correlation coefficient for each variable. Reliability was excellent for latency to orientation to the social stimulus (ICC = 0.98) and to the non-social stimulus (ICC = 0.99), excellent for the duration of looking time to the social stimulus (ICC = 0.91), and very good for the non-social stimulus (ICC = 0.87).

Dogs that were already orientated to the stimulus when the stimulus started to move were excluded from the latency to orientate analysis (Non-social: *N* = 17, Social: *N* = 20). We used linear mixed effects models with condition (Non-social vs. Social), age, order of conditions, lifelong training score, sex and neuter status as fixed effects and dog id as a random effect in the model. We included three-way interactions between breed, stimulus and age as well as between age, stimulus and lifelong training, and the two-way interactions between age and lifelong training in the model. Latency to orientation was inverse square root transformed and duration of looking at the stimuli was square transformed to fulfill the assumptions of normality and homogeneity of variance. We also calculated the correlation between duration of looking at the social stimulus and duration of looking at the non-social stimulus using Spearman’s rank correlation test, to examine whether dogs’ attentional performance was consistent across the two conditions.

### Results

**FIGURE 2:**
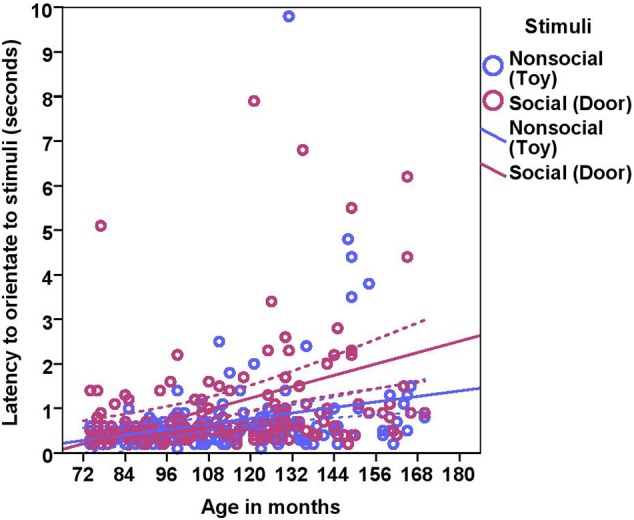
**Scatter plot showing the relationship between age in months and the latency to orientate to the social and the non-social stimuli [with 95% confidence intervals (dotted lines)]**. A significant effect of age in months (*r* = 0.41, *p* < 0.001) and stimuli (*p* < 0.001) was present on the latency to orientate to stimuli. One extreme data point was removed from this figure (latency to orientate: 19.8 s).

On average, dogs needed 0.70 s (range = 0.1–9.8 s, *SD* = 0.97) to orient to the non-social stimulus (toy) and 1.06 s (range = 0.20–19.8, *SD* = 1.86) to look toward the social stimulus (door). We found significant effects of age in months and stimuli on the orientation response (**Table 2** and **Figure 2**), Moreover, we found significant differences across the age groups with group2 being slower to orient than group1, and group3 slower than group1 and group2 in both conditions (**Table 2**). The removal of one outlier (latency to orientate = 19.8 s) did not change the results.

**Table 2:** Results of the linear mixed effects models on the two variables tested in attention test.

	Attention test						
Latency to orientate to stimuli	Estimate	Standard error	df	*t*-value	*p*-value	*r*	*R*^2^
Stimuli	–0.223	0.045	171.50	–4.981	**<0.001**	0.356	0.126
Age in months	–0.006	0.001	169.20	–5.982	**<0.001**	0.418	0.175
Breed	–0.082	0.050	171.90	–1.623	0.106	0.123	0.015
Training	–0.003	0.003	167.10	–1.067	0.288	0.082	0.007
Age_group							
Group1-Group2	–0.143	0.062	176.13	–2.293	**0.023**	0.170	0.029
Group2-Group3	–0.176	0.058	166.60	–3.031	**0.001**	0.229	0.052
Group1-Group3	–0.318	0.059	173.49	–5.375	**<0.001**	0.378	0.143
**Duration of looking at the stimuli**							
Stimuli	1297.685	90.452	161.54	14.347	**<0.001**	0.749	0.560
Age in months	–7.594	2.009	180.04	–3.779	**<0.001**	0.271	0.073
Breed	–121.331	104.783	180.04	–1.158	0.248	0.086	0.007
Training	19.071	6.481	178.82	2.943	**<0.01**	0.215	0.046
**Age_group**							
Group1-Group2	–25.610	126.170	178.59	–0.203	0.839	0.015	0.000
Group2-Group3	–388.000	120.290	178.48	–3.226	**<0.01**	0.235	0.055
Group1-Group3	–413.610	121.050	179.19	–3.417	**<0.001**	0.247	0.061

*Significant results (*p* < 0.05) are highlighted in bold letter.*

While BC and other breeds slightly differed in their average latency to orientate to the non-social stimulus [BC: 0.52 s (range = 0.1–1.50 s, *SD* = 0.29), other breeds: 0.81 s (range = 0.2–9.80 s, *SD* = 1.20)] and the social stimulus [BC: 0.71 s (range = 0.20–4.0 s, *SD* = 0.57), other breeds: 1.37 s (range = 0.20–19.8 s, *SD* = 2.37)], we found no effects of breed group or lifelong training. The results of the equivalence analysis in **Table 4** showed that the 90% confidence interval is not contained within the equivalency interval and therefore the two breed groups are not equivalent using the 10% criteria.

**FIGURE 3:**
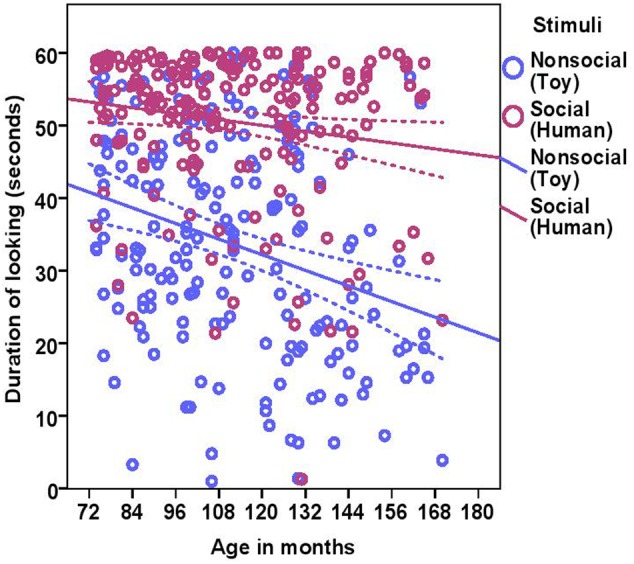
**Scatter plot showing the relationship between age in months and duration of looking at the social and the non-social stimuli [with 95% confidence intervals (dotted lines)].** A significant effect of age in months (*r* = 0.27, *p* < 0.001) and stimuli (*p* < 0.001) was present on the duration of looking at the stimuli.

**FIGURE 4:**
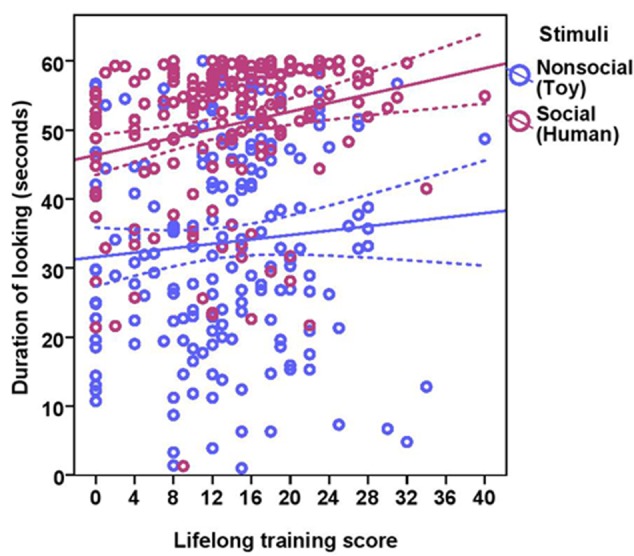
**Scatter plot showing the effect of lifelong training on duration of looking at the social and the non-social stimuli [with 95% confidence intervals (dotted lines)].** A significant effect of lifelong training (*r* = 0.21, *p* < 0.01) was present on the duration of looking at the stimuli.

The total duration of looking at the stimulus was significantly higher for the social stimulus compared to the non-social stimulus (human = 50.58 ± 10.34, toy = 33.72 ± 14.89; see **Table 2** for statistical results; **Figure 3**) and these two were weakly correlated with each other (Spearman’s rho = 0.15, *p* = 0.03). Duration of looking decreased with age in months both in the social and non-social conditions (**Table 2** and **Figure 3**) and we found significant differences across age groups. There was no difference between group1 and group2; however, group3 had a significantly lower duration of looking compared to group1 and group2. There was a significant effect of lifelong training, with dogs with a high training score looking at the stimulus for a longer duration compared to dogs with a low training score (**Table 2** and **Figure 4**).

While BC and other breeds slightly differed in their average duration of looking at the non-social stimulus [BC: 34.08 s (range = 1–57.10 s, *SD* = 15.01), other breeds: 33.47 s (range = 3.90–60.0 s, *SD* = 14.87)] and the social stimulus [BC: 53.13 s (range = 21.60–60.0 s, *SD* = 8.49), other breeds: 48.84 s (range = 1.30–60.0 s, *SD* = 11.13)], we found no effect of breed group (**Table 2**). The results of the equivalence analysis (in **Table 4**) showed that the 90% confidence interval is not contained within the equivalency interval and therefore the two breed groups are not equivalent using the 10% criteria.

### Discussion

In Experiment 1, we examined the effects of age, lifelong training and breed on attentional capture and sustained attention across three age groups of dogs using a very simple attention task that did not require prior training. The results revealed that: (a) the dogs’ ability to orientate to the stimuli showed a significant relationship with age; senior and geriatric dogs were slower to orient to the stimuli compared to dogs in late adulthood, (b) sustained attention also decreased with age, with geriatric dogs showing a major decline, (c) dogs with a high lifelong training score showed increased sustained attention, (d) dogs looked longer at the social stimulus (human) compared to the non-social stimulus (toy), and (e) no breed effect was found in any measures of attention.

Human studies measuring attentional capture suggest that control over attentional capture declines during normal aging, which possibly explains the slower orientation to the stimuli shown by older dogs in our study (Zanto and Gazzaley, 2014). However, the simpler explanation for general slowing as age increases is sensory-processing difficulties, which are inherent to advancing age both in humans (Staub et al., 2013) and dogs. The latency to orientation was higher for the social stimulus compared to the non-social stimulus, which can be explained by the proximity of the stimuli to the dog. The toy was closer to the dog than the door so when the toy moved, dogs were faster to orient.

Regarding sustained attention, the total duration of looking at the non-social stimulus (toy) and social stimulus (human) both decreased with age. Geriatric dogs sustained their attention to the toy and human to a lesser extent than senior and late adulthood dogs. In the human literature, there are two theories that explain the aging of sustained attention: (1) the resource depletion or mental fatigue theory and (2) the mindlessness or boredom theory. The resource depletion theory proposes that the decrement in sustained attention is caused by a decline of available attentional resources over time on the task. The mindlessness theory instead argues that the failure of sustained attention is caused by the repetitive, monotonous and non-arousing nature of the task, and thereby decreases in endogenous attentional control as the task advances (for details see Staub et al., 2013). Although sustained attention tasks applied to humans last much longer than 1 min, both mechanisms may play a role in the declining attention in older dogs found in this study. Alternatively, it is also possible that the older dogs had had more experience with various everyday events in their lives where they had learnt to focus their attention on relevant stimuli and to disregard irrelevant stimuli such as moving objects (Wallis et al., 2014; Mongillo et al., 2016). In addition to these explanations, habituation to the specific stimuli might also be an important contributor to decreased attention in geriatric dogs. Habituation occurs when an organism reduces or ceases its response to a specific stimulus after prolonged or repeated exposure to that stimulus (Eckel-Mahan and Sassone-Corsi, 2010). Response to novelty and habituation have been found to change with age in humans and rats (Kondratova et al., 2010). Similarly, we cannot exclude that older dogs habituated faster than younger ones, although the stimuli in this study were presented only for a short duration.

The difference found in sustained attention to the social and non-social stimuli can be due to multiple factors, including the salience of the stimuli, their biological relevance, and the animals’ motivation as well as cognitive ability to pay and maintain attention (Mongillo et al., 2016). Since we did not measure the motivation of dogs toward either of the stimuli we presented, it is possible that the dogs were differently motivated to look at the toy and the human because of their prior experiences with such stimuli. However, it is difficult to select stimuli that will be equally motivating to all dogs since every individual may have different experiences. A more consistent explanation for this finding may be that all dogs had gained positive experiences with the experimenter in the previous tests of the test battery, which could have motivated the dogs to attend to her more than to the novel non-social object. Other studies have also showed that positive reinforcement during previous training or other interactions is highly correlated with levels of attention (Lindsay, 2001; Horn et al., 2013).

We found a positive effect of lifelong training on sustained attention in dogs. Dogs that had a high lifelong training score sustained their attention for longer compared to dogs with a low lifelong training score or no previous training. Studies in humans have revealed positive effects of physical activity and cognitive training on enhancing different cognitive functions including attention (Shatil, 2013). Recent studies in pet dogs have provided some examples of the positive effect of lifelong training on performance in different cognitive tasks (Marshall-Pescini et al., 2008, 2009, 2016; Range et al., 2009). Studies in rats and laboratory beagles have also documented the effect of environmental enrichment, cognitive training and physical activity in improving cognitive performance (Milgram et al., 2005; Kramer et al., 2006; Nippak et al., 2006; Berchtold and Cotman, 2009; Harati et al., 2011; Snigdha et al., 2014). Hence, the dogs that had participated in different forms of training in their life might have benefited from those training experiences, and thus showed increased attention.

Border collies and other breeds did not differ in attentional capture and sustained attention, which indicates that the aging trajectories of the various components of attention seem to be consistent across breeds, despite different breeds having differing median lifespans (O’Neill et al., 2013). Mehrkam and Wynne (2014) also mentioned that breed differences are reported more in certain temperament traits rather than in cognitive abilities, which is in line with our findings. Although we found no significant differences between the breed groups, we could not confirm the equivalency of the two groups, BC and other breeds. Therefore, we cannot exclude the possibility that the power to detect the differences between BC and other breeds could be low (but see discussion of equivalency tests below).

## Experiment 2: Clicker Training For Eye Contact

The most common method used to measure selective attention in humans is the visual search task, which requires participants to attend to a target stimulus while ignoring distracters that are presented simultaneously. Additionally, the task may also involve response switching between tasks, which increase the task difficulty, leading to differences in younger and older individuals. Measuring selective attention in a task, where the dogs had to switch between establishing eye contact with the experimenter and finding food dropped on the floor, Wallis et al. (2014) reported that selective attention peaked at middle ages (>3–6 years) in BC. Using the same paradigm, we tested for the effect of breed, lifelong training and age, with the prediction that the dogs will show a decline of selective attention with old age.

### Procedure

**FIGURE 5:**
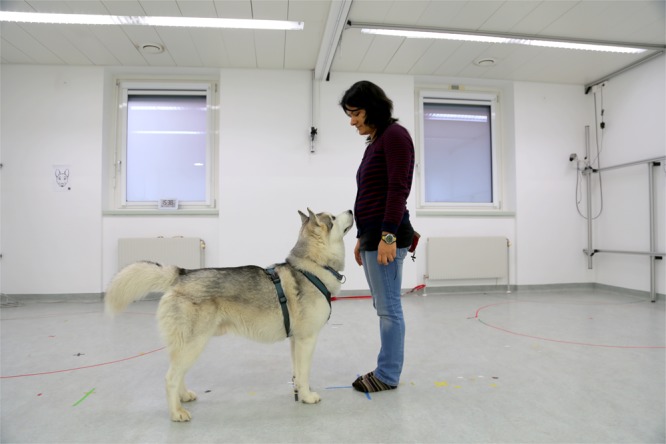
**Dog establishing eye contact with the experimenter**.

The owner entered the experimental room with her dog on a leash, released the dog, sat down on a chair positioned at the back wall of the experimental room and pretended to read the protocol. E stood in the center of the room with her back turned to the owner, holding a clicker in her right hand while her other hand was free. She had a food pouch containing sausage cut into <1 cm^3^ pieces on her belt, positioned at her back (**Figure 5**). For the first trial, she called the dog’s name and threw a piece of sausage on the floor for the dog to find. She then waited for the dog to establish eye contact with her after it had found and eaten the food, whereupon she clicked a clicker, threw again a piece of sausage on the floor and then again waited for the dog to establish eye contact after it had found and eaten the sausage. If the dog showed no interest and wandered away from E, she rustled the plastic bag containing the sausage for few seconds in order to call the attention of the dog, and then again remained motionless until the dog established eye contact with her. The experimenter continued this task for a total of 5 min.

### Statistical Analyses

In this test, we used the latency to eye contact (LEC) with the experimenter (measured from the moment the dog had taken the sausage into its mouth until the dog looked up into E’s face, which was marked by a click from the clicker), and the latency to find food (LFF; measured from the moment the piece of sausage left the E’s hand, until the dog had found and taken the sausage into its mouth) as measures of selective attention and sensorimotor control. The dogs’ initial performance in the task was measured by taking the average of the first three trials in both LEC and LFF. We also assessed learning across trials by comparing the average of the first three and the last three trials. A randomly chosen set of 20 dogs were double coded independently by a second coder and inter observer reliability was calculated by estimating the intra-class correlation coefficient for each variable. Reliability was excellent for LEC in the first three and last three trials (ICC = 0.99 and ICC = 0.99), and very good for latency to find food in the first three and last three trials (ICC = 0.86 and ICC = 0.96).

Five dogs were excluded from the analysis of initial performance in LEC and latency to find food, since they did not manage to complete three trials in the allotted 5 min time limit. To calculate the average latency of the last three trials, we set the criterion to a maximum of 20 trials for each dog, i.e., for dogs that had more than 20 trials within 5 min; LEC and LFF were taken from trials 18, 19, and 20. Accordingly, only dogs that had completed at least six trials during the 5-min test were included in the calculation of the average of the last three trials. Twelve dogs were excluded because they did not reach this criterion. We calculated a difference score by subtracting the average of the first three trials and the average of the last three trials for every dog, and ran the analysis to examine task specific learning across trials.

The data were analyzed using linear models with age, lifelong training score, sex, neuter status and previous clicker experience (yes/no) as fixed effects. We also included the two-way interactions between age and breed, age and lifelong training, and breed and lifelong training in the model. LEC was inverse square root transformed and LFF was inverse-transformed to fulfill the assumptions of normality and homogeneity of variances. When analyzing the difference between the average of the first three trials and the last three trials, LEC was power transformed (Boxcox, lamda = -0.34). It was not possible to normalize the data to calculate task-specific learning across trials for LFF; therefore, we used non-parametric statistics (Wilcoxon-sign rank test). We also calculated the correlation between LEC and LFF using Spearman’s rank correlation test.

### Results

**FIGURE 6:**
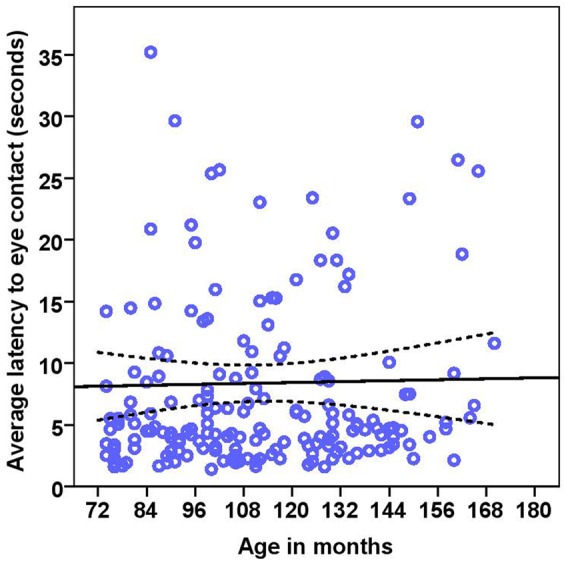
**Scatter plot showing relationship between age in months and the latency to eye contact with the experimenter [with 95% confidence intervals (dotted lines)]**. No effect of age in months (*r* = 0.08, *p* = 0.285) was seen on the latency to eye contact. Two extreme data points were removed from this figure.

**FIGURE 7:**
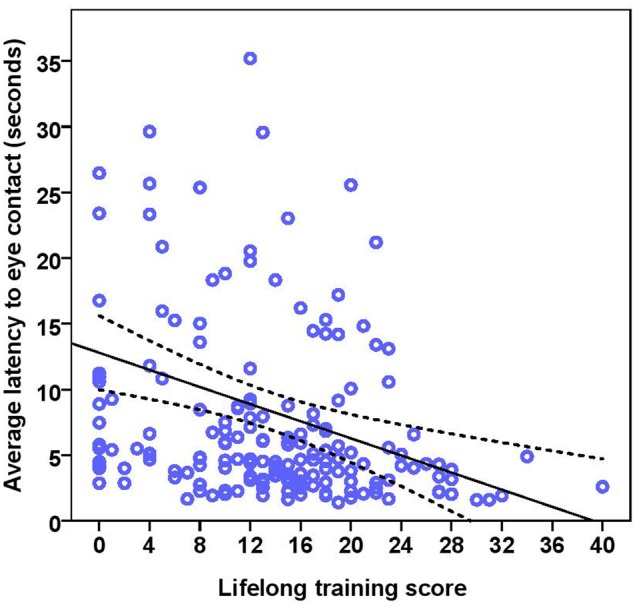
**Scatter plot showing the effect of lifelong training on the latency to eye contact with the experimenter [with 95% confidence intervals (dotted lines)].** A significant effect of lifelong training (*r* = 0.30, *p* < 0.001) was present on the latency to eye contact. Two extreme data points were removed from this figure.

The dogs’ LEC with the experimenter was on average 8.37 s (range = 1.40–77.43 s, *SD* = 9.96 s) in the first three trials. There was no effect of age (see **Table 4** for statistical results, **Figure 6**) or breed group on LEC. Significant effects of lifelong training score (**Figure 7**) and clicker experience was found. Dogs with a higher lifelong training score and clicker trained dogs were faster to establish eye contact with the experimenter than dogs with lower lifelong training and clicker naive dogs. When using age group as a predictor, no significant age differences were found. While BC were slightly faster (7.22 s; range = 1.40–29.57 s, *SD* = 5.77) than other breeds (9.17 s; range = 1.60–77.43 s, *SD* = 12.01) to establish eye contact with the experimenter, this difference did not reach statistical significance. The results of the equivalence analysis (in **Table 4**) showed that the 90% confidence interval is not contained within the equivalency interval and therefore the two breed groups are not equivalent using the 10% criteria.

**FIGURE 8:**
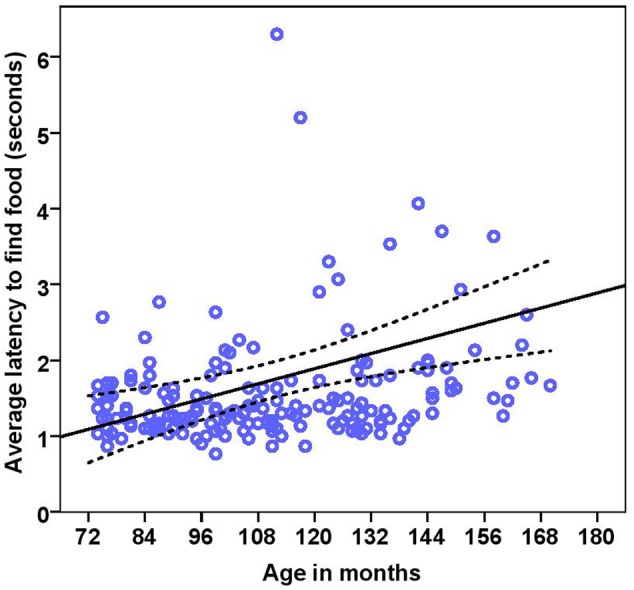
**Scatter plot showing relationship between age in months and the latency to find food [with 95% confidence intervals (dotted lines)].** A significant effect of age in months (*r* = 0.27, *p* < 0.001) was present on the latency to find food. Two extreme data points were removed from this figure.

**FIGURE 9:**
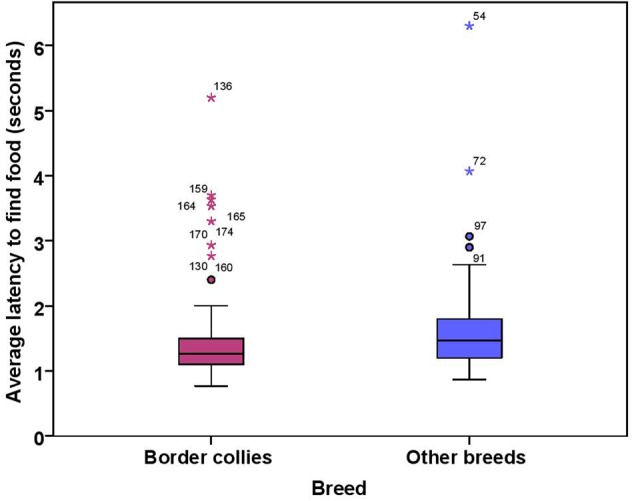
**Box plot showing effect of breed group on the latency to find food.** Difference between two groups was significant (*p* < 0.01). Two extreme data points were removed from this figure.

The dogs’ latency to find food in the first three trials was on average 1.74 s (range = 0.77–15.23 s, *SD* = 1.64). BC on average took 1.49 s (range = 0.77–5.20 s, *SD* = 0.76) while other breeds took 1.92 s (range = 0.87–15.23 s, *SD* = 2.03) to find food on the floor. There were significant effects of age, breed group and clicker experience, with older and mixed breed dogs needing longer to find food than younger dogs and BC, and clicker trained dogs having a shorter latency to find food than non-clicker trained dogs (see **Table 3** for statistical results, **Figures 8, 9**). There was a significant effect of age group, where group 3 differed from group 1 and group 2, but no difference was found between group 1 and group 2. Interestingly, lifelong training score had no effect on the latency to find food. The latency of dogs to establish eye contact was significantly positively correlated with their latency to find food (Spearman’s rho = 0.275, *p* < 0.001).

**Table 3:** Results of linear models on the two variables tested in clicker training for eye contact task.

	Clicker training for eye contact						
Latency to eye contact	Estimate	Standard error	df	*t*-value	*p*-value	*r*	*R*^2^
Training	0.006	0.001	175	4.099	**<0.001**	0.296	0.088
Clicker_experience	0.092	0.023	175	4.010	**<0.001**	0.290	0.084
Age in months	0.000	0.000	175	–1.073	0.285	0.081	0.007
Breed	0.016	0.023	175	0.681	0.497	0.051	0.003
**Latency to find food**							
Training	–0.002	0.002	175	–1.187	0.237	0.089	0.008
Clicker_experience	0.087	0.032	175	2.716	**<0.01**	0.201	0.040
Age in months	–0.002	0.001	175	–3.749	**<0.001**	0.273	0.074
Breed	–0.100	0.032	175	–3.118	**<0.01**	0.229	0.053
**Age_group**							
Group1-Group2	–0.018	0.039	174	–0.453	0.651	0.034	0.001
Group2-Group3	–0.091	0.038	174	–2.418	**0.001**	0.180	0.033
Group1-Group3	–0.108	0.038	174	–2.875	**<0.001**	0.213	0.045

*Significant results (*p* < 0.05) are highlighted in bold letter. The removal of two outliers (LEC = 77.43 s and 70.60 s; LFF = 11.23 s and 12.60 s) did not change the results.*

**Table 4:** Equivalency test results of Border collies vs. other breeds for the different attention variables.

Variables		90% CI		
	Equivalence criterion^a^	LCL	UCL	*p*-value^b^
Latency to orientate to non-social stimulus	±0.052	–0.501	–0.089	0.974
Latency to orientate to social stimulus	±0.071	–1.078	–0.253	0.991
Duration of looking at non-social stimulus	±3.4	–2.86	4.62	0.134
Duration of looking at social stimulus	±5.31	1.884	6.691	0.240
Latency to eye contact	±1.44	–4.189	0.285	0.819

*^a^Criterion is ± 10% of the Border collie group mean. ^b^The *p-*value of the two one-sided tests, *p <* 0.05 for equivalency. CI, confidence interval; LCL, lower confidence limit; UCL, upper confidence limit.*

When comparing the LEC in the last three trials to the first three trials, results showed that the dogs’ LEC significantly decreased during the clicker training task [mean LEC first 3 trials = 7.95 s vs. mean LEC last 3 trials = 5.91 s; estimate ± SE = -0.20 ± 0.02, *t*_(170.17)_ = -7.58, *p* < 0.0001]. However, latency to find food did not change over the trials (mean LFF first 3 trials = 1.65 s vs. mean LFF last 3 trials = 1.61 s, *Z* = -1.14, *p* = 0.25).

### Discussion

In Experiment 2, we investigated the effects of age, lifelong training and breed on a selective attention task, where dogs had to switch between two tasks, forming eye contact with the experimenter and finding food on the floor. The results showed that there was no effect of age or breed group, but a significant effect of lifelong training on dogs’ LEC, and a significant effect of age and breed, but no effect of lifelong training on dogs’ latency to find food.

Selective attention requires the simultaneous presentation of two or more stimuli of which one or more are distractors (Grilly et al., 1989), and among which the subject is required to locate the correct object (Snigdha et al., 2012). Additionally, the task may also involve response switching between tasks, which increase task difficulty. Task difficulty pays a crucial role in influencing selective attention. While we used a task switching paradigm, we only switched between two responses, which might have been perceived as too simple by the dogs, masking any age effects in this task. In a more complex task, where two or more (distracting) stimuli were presented, aged laboratory dogs showed a deficit in selective attention (Snigdha et al., 2012). Similarly, in a social version of the task when the dogs had to choose between looking at their owner or a stranger who were presented simultaneously, old dogs showed a decline in selective attention by looking more at the stranger in comparison to younger dogs (Mongillo et al., 2010). Thus, future studies should attempt to use tasks involving different distractors and multiple responses switching in order to increase task difficulty, and to confirm if age effects on selective attention parameters are present in pet dogs.

We found a significant effect of lifelong training on the latency to form eye contact with the experimenter. It seems understandable given that every type of dog training begins with a learning to look at the handler (Mongillo et al., 2016). Thus, dogs with a high lifelong training score might have transferred their prior knowledge to the experimental training context and therefore were faster to form eye contact. Alternatively, the training effect could also be driven by clicker training experience; since there was also a positive effect of clicker experience on the lifelong training score, and additionally clicker trained dogs were faster to form eye contact with the experimenter than non-clicker trained dogs. So it is noteworthy to mention that clicker training seems to improve human directed looking behavior and also increases the motivation to attend to the experimenter/handler in new training contexts. Dogs improved their performance across the 20 training trials and the learning effect was clearly evident, as the mean latency of the last three trials was significantly lower than the first three trials. All dogs were able to significantly reduce their latencies. Since dogs were rewarded for forming eye contact with the experimenter by a click and throwing food on the floor for 5 min, this repeated in-task training increased the motivation of all dogs regardless of their age to allocate their attention to the experimenter more quickly as the time elapsed.

The second variable we measured in Experiment 2 was latency to find food, and in accordance with our prediction, there was a significant effect of age. Dogs were slower to find food as age increased; geriatric dogs were the slowest of all the age categories. Deficiencies in finding food can simply be explained by the physical deterioration of visual, auditory or olfactory organs, or reduced sensorimotor capability inherent to advancing age (Wallis et al., 2014). Other possible explanations include alterations in the cognitive processing of sensory information, reduced cognitive resources or increased distractibility, all of which were suggested as possible reasons for the inferior performance of older dogs in selective attention tasks (Tapp et al., 2003; Mongillo et al., 2010; Snigdha et al., 2012). Previous studies have reported that older humans are more prone to distraction than young ones, and also show reduced ability to filter out irrelevant information. Increased distractor processing reflects an age-related decline in a central inhibitory mechanism (Ballesteros and Mayas, 2015). Similar mechanisms might be involved in both humans and pet dogs in regulating selective attention. It can be argued that the older dogs have a lower motivation to find the food; however, motivational differences are unlikely because we did not find any age effects on the initial latency to form eye contact. All dogs were highly motivated to attend to the experimenter, and additionally, dogs’ performance remained consistent over 20 trials of finding food. Difficulty in finding dropped food on the floor was one of the most consistent findings in old dogs during normative aging, as documented in a cognitive aging questionnaire (Salvin et al., 2009). The fact that geriatric dogs took longer to find dropped food in our study provides an objective measurement for the result reported via the cognitive aging questionnaire. Although there was no effect of lifelong training on latency to find food, clicker trained dogs were quicker to find food than non-clicker trained dogs, which simply suggests that this kind of task was already familiar to clicker trained dogs, and clicker training in general seems to increase the anticipation of receiving food.

Border collies and other breeds of dogs differed only in latency to find food in this task. Since BC display eye-stalk-chase motor patterns during herding, subtle movements of the experimenter while throwing food on the floor might have been a clearer cue for them to be quick to look for the food than other breeds. Although we found no differences between breed groups in latency to establish eye contact, we could not confirm the equivalency of BC and other breeds. Therefore, we cannot exclude the possibility that the power to detect the differences between BC and other breeds could be low (see discussion of equivalency tests below).

## General Discussion and Conclusion

We investigated the aging of attentiveness in pet dogs and found that different attention functions are affected differentially during aging. Similarly to aged humans, geriatric dogs showed the largest decrease in attentional capture and sustained attention compared to other age categories. In contrast, selective attention showed no decline in aged dogs, as it does in humans. Our findings on attention, in addition to several other studies examining cognitive aging in dogs (Milgram et al., 1994; Adams et al., 2000; Tapp et al., 2003; Tapp, 2004) add to the growing body of evidence that the domestic dog is a suitable model for human cognitive aging. Importantly, by using pet dogs as a model of human cognitive aging, we are able to generalize the findings obtained from the laboratory settings into real life environmental settings, which will facilitate the development of preventive and treatment strategies to delay cognitive decline in both dogs and humans. Cognitive aging studies in pet dogs also enable us to examine different interventions, such as training, that could influence attentiveness in a positive way.

Our study revealed that lifelong training had a positive effect on sustained attention and selective attention. Lifelong training including clicker training seems to be an effective intervention for retaining attentiveness and preventing the aging of attentiveness in pet dogs Research on humans has focused on unraveling both the separate and combined effects of physical activity and cognitive training on cognition in older adults. These studies have revealed larger benefits on cognitive performance from combined physical and cognitive activity rather than for each activity alone (Bamidis et al., 2014). Physical activity and cognitive training may induce both temporary and permanent changes at the structural and functional levels in the aging brain. The protecting effects of physical activity and cognitive training on cognitive decline have also been supported by neuroscientific evidences (Bamidis et al., 2014). In dogs, there is some evidence for a positive effect of lifelong training on dogs’ problem solving abilities (Marshall-Pescini et al., 2008, 2009, 2016; Range et al., 2009), which could presumably be explained by heightened attention in trained dogs. Hence, the positive effect of lifelong training that we observed in our study for several attention parameters, adds to the existing evidence that lifelong training can delay the aging of attentiveness.

Border collies and other breeds did not differ, but were also not equivalent regarding the measures of attentional capture and sustained attention. We estimated the “equivalence” or “non-equivalence” of other breed dogs with reference to the Border collie sample. Equivalence analysis makes it possible to determine whether differences that are not statistically significant may be the consequence of small sample sizes or large variability rather than an indication of actual equivalence between the groups being compared (Leff et al., 2005). Our results showing that BC and other breeds were not different and not equivalent indicates that the study is underpowered for those particular variables measured (Leff et al., 2005). Therefore, the lack of differences that we obtained in our results might be merely due to lack of statistical power, possibly because of the combination of large differences between the standard deviations of the population means and a small magnitude of the true difference. In all variables measured, the group “other breeds” had a higher standard deviation than BC. This is to be expected as the other breed group contained a total of 30 different breeds, including mixed breeds, compared to only one breed in the Border collie group. However, there was a considerable overlap between the two groups in variable range. Calculations using the observed means and SD revealed that in order to detect a minimal statistical difference between the populations (effect size = 0.33, power of 0.95), the sample size would have to be around 240 individuals in each group. Future studies should aim to examine and compare additional individual breeds to confirm the absence of breed differences in attention in aged pet dogs.

Overall, our study provided short simple tasks designed to measure sustained and selective attention in dogs in naturalistic situations. Compared to tests previously applied to laboratory beagles, our tasks are more effective because they do not require prior task-training experience and no training is necessary during the task. Therefore, by utilizing these tasks, attentional changes during aging in pet dogs can be evaluated quickly and efficiently. In summary, our results of the aging of attentiveness in pet dogs complement findings in aged laboratory beagles and older humans, which in turn highlight the utilization of dogs as a model species for studying cognitive aging.

## Author Contributions

Conceived and designed the experiments: DC, ZV, FR, and LW. Performed the experiments: DC. Analyzed the data: DC, ZV, FR, and LW. Interpretation of results: DC, FR, ZV, LW, and LH. DC wrote first draft of paper. Revising the paper: DC, FR, LW, ZV, LH, and JS. Providing funding: JS, ZV, and FR. Other (video coding): DC and LW.

## Conflict of Interest Statement

The authors declare that the research was conducted in the absence of any commercial or financial relationships that could be construed as a potential conflict of interest.

## Footnotes

1 http://solomoncoder.com
