# Supplementary material for: Aging of Attentiveness in Border Collies and Other Pet Dog Breeds: The Protective Benefits of Lifelong Training
Source: Front Aging Neurosci. 2017 Apr 20;9:100. doi: 10.3389/fnagi.2017.00100 (PMC5397477; doi:10.3389/fnagi.2017.00100)
Supplement: Supplementary file 1 [file Presentation_1.PDF]

Supplementary Material

Methods:

As mentioned in the subjects section of the manuscript, before adding 16 new BC to the 59 BC tested by Wallis et al. (2014), we analysed all the variables to see whether the new BC sample differed from Wallis BC. For this, a subset of 16 BC matched for age, sex and lifelong training score to that of the 16 new BC were selected from Wallis 59 BC sample and the analysis was run to see whether the two groups of BC differ from each other in any of the variables measured in experiment 1 and 2. Statistical analyses were performed in R 3.2.2 (RStudio 2015). For the variables, latency to orientate to the stimuli and duration of looking, we used linear mixed effects models with age, stimulus (social/non-social) and study (Chapagain/Wallis) as fixed effects and dog identity as a random factor. For latency to eye contact and latency to find food, we used linear models with age, training and study as fixed effects in the model. Normality and homoscedasticity were assessed via residual distribution charts and plots of residuals against fitted values. Latency to orientate to stimuli and latency to eye contact were inverse square root transformed, duration of looking was square transformed, and latency to find food on the floor was inverse transformed to fulfill the assumptions of normality and homogeneity of variance. Results are presented as means± SD.

Results:

Experiment1: Attention test

On average, Wallis BC needed 0.58 seconds (range= 0.2-1.40s, SD=0.31) to orient to the non-social (toy) and 0.68s (range=0.30-2.20s, SD=0.43) to look towards the social stimuli (human). Similarly, Chapagain BC needed 0.49s (range= 0.20-1.0s, SD=0.23) to orient to the non-social and 0.83s (range=0.20-4.0s, SD=0.88) to look towards the social stimuli. There was no significant difference between the orientation response of Wallis BC and Chapagain BC (estimate= -0.05 ±0.09, $t_{(29.18)}$= -0.55, p=0.60)

On average, Wallis BC looked at the non-social stimulus for 35.75 seconds (range= 3.30-52.30s, SD=17.14) and social stimulus for 53.85s (range=28.10-59.70s, SD=8.15). Similarly, Chapagain BC looked at the non-social stimulus for 27.40s (range= 1-55.50s, SD=17.51) and social stimulus for 49.06s (range=21.60-60s, SD=11.28). There was only a tendency for the difference between the duration of looking of Wallis BC and Chapagain BC to be significant (estimate= 475.55 ± 240.889, $t_{(29)}$ = 1.97, p=0.05).

Experiment 2:

Clicker training for eye contact:

The average time taken by Wallis BC to make eye contact with the experimenter was 5.90s (range=1.67-18.33s, SD=4.37). Similarly, Chapagain BC took 5.97s (range=1.4-15.27s, SD=4.48) to make eye contact with the experimenter. Regarding the average latency to find food, Wallis' BC took 1.42s (range=0.87-3.53s, SD=0.69) and Chapagain BC took 1.41s (range=1.07-2.77s, SD=0.44). The measures of latency to eye contact between Wallis' BC and Chapagain BC was not significantly different (estimate= -0.05 ± 0.05, $t_{(29)}=-1.03$, $p=0.31$). Similarly, latency to find food also did not differ between Wallis' BC and Chapagain BC (estimate= 0.05 ± 0.07, $t_{(28)}= 0.66$, $p=0.51$)

Discussion:

Since the two subsets of Border collies (Wallis BC and Chapagain BC) did not differ from each other, we decided to add the 16 new BC tested by Chapagain to the 59 BC tested by Wallis et al. (2014) for the current manuscript.
